# Development of a Reproducible Prognostic Gene Signature to Predict the Clinical Outcome in Patients with Diffuse Large B-Cell Lymphoma

**DOI:** 10.1038/s41598-019-48721-0

**Published:** 2019-08-21

**Authors:** Mohamad Zamani-Ahmadmahmudi, Seyed Mahdi Nassiri

**Affiliations:** 1 Department of Clinical Science, Faculty of Veterinary Medicine, Shahid Bahonar University of Kerman, Kerman, Iran; 2 Department of Clinical Pathology, Faculty of Veterinary Medicine, University of Tehran, Tehran, Iran

**Keywords:** Lymphoma, Non-hodgkin lymphoma

## Abstract

Alongside various clinical prognostic factors for diffuse large B-cell lymphoma (DLBCL) such as the international prognostic index (IPI) components (ie, age, tumor stage, performance status, serum lactate dehydrogenase concentration, and number of extranodal sites), prognostic gene signatures have recently shown promising efficacy. However, previously developed signatures for DLBCL suffer from many major inadequacies such as lack of reproducibility in external datasets, high number of members (genes) in a signature, and inconsistent association with the survival time in various datasets. Accordingly, we sought to find a reproducible prognostic gene signature with a minimal number of genes. Seven datasets—namely GSE10856 (420 samples), GSE31312 (470 samples), GSE69051 (157 samples), GSE32918 (172 samples), GSE4475 (123 samples), GSE11318 (203 samples), and GSE34171 (91 samples)—were employed. The datasets were randomly categorized into training (1219 samples comprising GSE10856, GSE31312, GSE69051, and GSE32918) and validation (417 samples consisting of GSE4475, GSE11318, and GSE34171) groups. Through the univariate Cox proportional hazards analysis, common genes associated with the overall survival time with a *P* value less than 0.001 and a false discovery rate less than 5% were identified in 1219 patients included in the 4 training datasets. Thereafter, the common genes were entered into a multivariate Cox proportional hazards analysis encompassing the common genes and the international prognostic index (IPI) factors as covariates, and then only common genes with a significant level of difference (*P* < 0.01 and z-score >2 or <−2) were selected to reconstruct the prognostic signature. After the analyses, a 7-gene prognostic signature was developed, which efficiently predicted the survival time in the training dataset (*Ps* < 0.0001). Subsequently, this signature was tested in 3 validation datasets. Our signature was able to strongly predict clinical outcomes in the validation datasets (*Ps* < 0.0001). In the multivariate Cox analysis, our outcome predictor was independent of the routine IPI components in both training datasets (*Ps* < 0.0001). Furthermore, our outcome predictor was the most powerful independent prognostic variable (*Ps* < 0.0001). We developed a potential reproducible prognostic gene signature which was able to robustly discriminate low-risk patients with DLBCL from high-risk ones.

## Introduction

Diffuse large B-cell lymphoma (DLBCL) as the most common type of lymphoma in adults accounts for approximately 30% of all cases of lymphoma^1,2^.

Development of prognostic gene signatures was started in a study conducted by Alizadeh *et al*.^3^, who proposed 2 distinct subtypes of DLBCL (ie, germinal center B cell-like [GCB] and activated B cell-like [ABC]) based on gene expression profiling. The authors indicated that the overall survival (OS) time was significantly higher in patients with GCB-DLBCL than in those with ABC-DLBCL. In a study by Rosenwald *et al*.^4^, another molecular subtype of DLBCL (type 3), which did not express the gene characteristics of either GCB or ABC DLBCL, was added to the previous subtypes. In addition, the authors proposed a 17-gene signature which could predict OS after chemotherapy. Via gene expression profiling and supervised machine learning, a 13-gene predictive model was reconstructed in 58 patients with DLBCL^5^. Surprisingly, their results revealed that the clinical outcome was not significantly different between 2 groups of patients based on the 90-gene model proposed by Alizadeh *et al*.^3^. Via a statistical method based on Bayes’ rule, a classifier comprising 27 genes was developed to subtly assign patients with DLBCL to ABC and GCB subgroups and a concluding 14-gene model was proposed as the final subgroup predictor^6^. Lossos *et al*.^2^, seeking to develop a predictive model using prognostic genes previously identified as single prognostic genes or as a member of prognostic signatures, suggested a 6-gene model among 36 genes as the final prognostic signature. Finally, a 108-gene model was created using a combination of 3 gene-expression signatures—namely “germinal-center B-cell,” “stromal-1,” and “stromal-2”—by Lenz *et al*.^7^. This large signature could predict survival in CHOP-treated or R-CHOP treated patients.

Despite the introduction of various prognostic gene signatures, there are still many disadvantages curtailing the clinical use of these signatures. Indeed, the most salient disadvantage of the previously developed signatures is lack of reproducibility in various datasets, with many of the genes in the proposed prognostic signatures failing to show a significant association with survival in external validation analyses (See the Results.) Furthermore, our analysis showed that many of these genes failed to exhibit a consistent prognostic pattern in different datasets as some genes with positive associations with the survival time in a dataset were negatively associated with survival in another dataset (See the Results). In addition, some of these signatures are considerably large and contain large numbers of genes (90 genes, 27 genes, and 180 genes in signatures developed by Alizadeh *et al*.^3^, Wright *et al*.^6^, and Lenz *et al*.^8^, respectively), rendering the clinical application of such large signatures difficult or impossible. Moreover, these developed signatures have shown minimal common genes with each other. For example, there were no common genes in the models derived by Shipp *et al*. (2003) and Rosenwald *et al*.^4^. Similarly, *BCL6* is the only common gene between signatures developed by Losses *et al*.^2^, Rosenwald *et al*.^4^, and Wright *et al*.^6^.

As another disadvantage, some of these studies used old microarray platforms, which might not be compatible with new platforms. For instance, some genes in Lymphochip-spotted cDNA microarrays^3,4^ cannot be found in new Affymetrix arrays.

Given all the above mentioned problems, we endeavored to develop a reproducible prognostic gene signature with a minimal number of genes using a strict pipeline in patients with DLBCL. Accordingly, using 4 training datasets, we identified common genes associated with the OS time in 1219 patients through stringent criteria. We reconstructed a prognostic signature with the extracted common genes and validated it externally in 417 patients included in 3 validation datasets. Finally, we produced a reproducible 7-gene signature, which was significantly associated with the survival time in both training and validation datasets and was by far the most powerful independent prognostic factor in comparison with the prognostic components of the IPI.

## Results

### Extraction of the common genes associated with survival and the reconstruction of the prognostic signature

First, search was conducted to find the common genes associated with survival between the 4 training datasets, encompassing 1219 patients. Our analysis revealed that 12 genes consistently had significant associations with OS at a *P* value less than 0.001 and an FDR less than 5% in all the datasets (Supplementary Table 1). The common genes included *APOC1*, *C5orf30*, *CALD1*, *CD84*, *CSF2RA*, *GPNMB*, *ITPKB*, *LPP*, *PDLIM4*, *PLAU*, *RTN1*, and *RGS3*. These genes showed consistent expression patterns in the 4 datasets, with 11 out of the 12 genes being positively associated with survival and the remaining gene (*C5orf30*) being negatively associated with survival (Supplementary Table 1). These genes also emerged as members in the class predictors developed using 2 different algorithms, which revealed that their expressions were significantly different between the 2 classes (long survival vs. short survival).

More robust and reliable findings were obtained by entering the common genes into the multivariate Cox analysis, where various components of the IPI and the common genes were considered as covariates. In this stage, only genes which reached a significant level were retained. Hence, genes with a *P* value less than 0.01 and a z-score greater than 2 or below −2 were selected to reconstruct the prognostic signature. Our analysis retained 7 genes—namely *APOC1*, *CALD1*, *CD84*, *GPNMB*, *ITPKB*, *PLAU*, and *RTN1*—and excluded 5 genes—namely *c5orf30*, *LPP*, *CSF2RA*, *PDLIM4*, and *RGS3* (Table 1). Although some genes such as *LPP*, *CSF2RA*, *PDLIM4*, and *RGS3* passed the defined criteria in 1 dataset, they did not reach a significant level in another one (Table 1). Hence, they were excluded for subsequent analysis.

**Table 1:** Analysis of multivariate Cox proportional hazards analysis of the common genes associated with survival time.

Gewne	Probe-set	Coefficient	HR^A^	z score	*P* value	Deleted
**GSE10846**						
*APOC1*	204416_x_at	−0.29	0.75	−3.2	0.0016	
*C5orf30*	221823_at	0.34	1.41	2.3	0.0217	Yes
*CALD1*	201615_x_at	−0.18	0.83	−3.8	0.0001	
*CALD1*	201616_s_at	−0.34	0.71	−4.2	0.0000	
*CALD1*	201617_x_at	−0.24	0.78	−3.6	0.0003	
*CALD1*	212077_at	−0.29	0.75	−3.2	0.0015	
*CALD1*	214880_x_at	−0.25	0.78	−2.9	0.0037	
*CD84*	211192_s_at	−0.17	0.84	−3.1	0.0020	
*CSF2RA*	207085_x_at	−0.20	0.82	−3.0	0.0031	Yes
*CSF2RA*	210340_s_at	−0.34	0.71	−4.9	0.0000	Yes
*CSF2RA*	211286_x_at	−0.24	0.78	−2.9	0.0036	Yes
*GPNMB*	1554018_at	−0.23	0.79	−3.4	0.0008	
*GPNMB*	201141_at	−0.34	0.71	−3.4	0.0006	
*ITPKB*	235213_at	−0.30	0.74	−4.2	0.0000	
*LPP*	202821_s_at	−0.20	0.82	−3.4	0.0007	Yes
*LPP*	202822_at	−0.13	0.87	−1.2	0.2170	Yes
*LPP*	224811_at	−0.07	0.93	−0.7	0.4820	Yes
*LPP*	235000_at	−0.24	0.79	−2.3	0.0216	Yes
*PDLIM4*	211564_s_at	−0.10	0.90	−1.9	0.0579	Yes
*PDLIM4*	214175_x_at	−0.09	0.92	−1.4	0.1698	Yes
*PLAU*	205479_s_at	−0.41	0.67	−2.7	0.0067	
*RGS3*	203823_at	−0.42	0.66	−2.7	0.0066	Yes
*RTN1*	203485_at	−0.27	0.76	−3.7	0.0003	
*RTN1*	210222_s_at	−0.25	0.78	−2.7	0.0072	
**GSE31312**						
*APOC1*	204416_x_at	−0.32	0.73	−4.0	0.000	
*C5orf30*	221823_at	0.07	1.07	0.6	0.521	Yes
*CALD1*	201616_s_at	−0.19	0.83	−2.5	0.013	
*CALD1*	201617_x_at	−0.32	0.72	−4.3	0.000	
*CALD1*	214880_x_at	−0.68	0.51	−4.7	0.000	
*CD84*	211192_s_at	−0.28	0.75	−2.9	0.004	
*CD84*	230391_at	−0.34	0.71	−3.7	0.000	
*CSF2RA*	207085_x_at	−0.02	0.98	−0.2	0.882	Yes
*CSF2RA*	210340_s_at	−0.11	0.89	−1.0	0.315	Yes
*CSF2RA*	211286_x_at	−0.10	0.90	−0.8	0.423	Yes
*GPNMB*	1554018_at	−0.24	0.78	−2.9	0.003	
*ITPKB*	235213_at	−0.26	0.77	−3.1	0.002	
*LPP*	202822_at	−0.19	0.83	−2.6	0.010	Yes
*LPP*	241879_at	−0.29	0.75	−3.9	0.000	Yes
*PDLIM4*	214174_s_at	−0.26	0.77	−3.0	0.003	Yes
*PLAU*	205479_s_at	−0.25	0.77	−3.2	0.003	
*PLAU*	211668_s_at	−0.38	0.69	−2.9	0.004	
*RGS3*	203823_at	−0.17	0.84	−1.2	0.227	Yes
*RTN1*	203485_at	−0.26	0.77	−3.5	0.000	

^A^Hazard ratio.

Genes with a *P* value < 0.01 and z score >2 or <−2 were selected to reconstruct prognostic signature.

**Figure 1:**
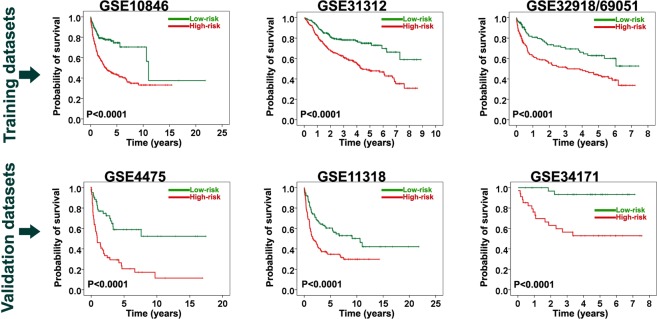
Kaplan-Meier survival analysis of the final 7-gene prognostic signature in the training and validation datasets. The final signature was found to be significantly associated with overall survival at a *P* value < 0.0001 in both training and validation datasets.

Finally selected 7 prognostic genes were used to reconstruct the prognostic gene signature as described in the method section. The patients in the training datasets were categorized into 2 groups based on this signature. As shown in Fig. 1, the survival time was significantly different between the low-risk and high-risk groups (*P* < 0.0001) in training datasets. In GSE10846, the rates of OS at 5 years in the low-risk and high-risk groups were 75% and 43%, respectively. Likewise, in GSE31312, the rates of OS at 5 years in the low-risk and high-risk groups were 75% and 48%, correspondingly. These value for low-risk and high-risk groups in GSE32918&69051 were 63% and 43%, respectively. The hazard ratio was significantly lower in the low-risk group than in the high-risk group in GSE10846 (HR = 0.39 [0.27–0.54]), GSE31312 (HR = 0.46 [0.33–0.63]) as well as in GSE32918&69051 (HR = 0.51 [0.35–0.75]) (*Ps* < 0.0001) (Table 2).

**Table 2:** Statistics of Cox proportional hazard analysis of the final prognostic signature in the training and validation datasets.

	*P* value	HR^A^	95% CI^B^
***Training datasets***			
GSE10846	**0**.**000**	0.39	0.27–0.54
GSE31312	**0**.**000**	0.46	0.33–0.63
GSE69051&32918	**0**.**000**	0.51	0.35–0.75
***Validation datasets***			
GSE4475	**0**.**000**	0.32	0.19–0.54
GSE11318	**0**.**001**	0.51	0.35–0.76
GSE34171	**0**.**000**	0.10	0.02–0.45

^A^Hazard ratio, ^B^Hazard ratio 95% confidence interval.

Significant *P* values were bolded.

Further analysis revealed that our developed prognostic signature was independent of routine IPI components in both training datasets (GSE10846: HR = 0.39 [0.26–0.59], GSE31312: (HR = 0.49 [0.34–0.72]) (*Ps* < 0.0001). Our outcome predictor was the most powerful prognostic variable in the multivariate Cox proportional hazards analysis (Table 3). Among the various components of the IPI, only age was able to predict the outcome in both datasets (*Ps* < 0.01) (Table 3).

**Table 3:** Multivariate analysis of the final prognostic signature and common prognostic variables in DLBCL (the IPI components).

Variable	GSE10846			GSE31312		
	*P* value	HR^A^	95% CI^B^	*P* value	HR	95% CI
Our signature	**0.000**	0.39	0.26–0.59	**0.000**	0.49	0.34–0.72
Sex (male vs. female)	0.677	0.9	0.64–1.3	0.15	1.3	0.91–1.8
Age (≥60 vs. <60 years)	**0.000**	2.0	1.4–3.1	**0.003**	1.7	1.2–2.4
**Molecular subtype**						
GCB-like vs. type 3	0.396	0.8	0.43–1.4	0.754	0.9	0.51–1.6
ABC-like vs. type 3	0.082	1.6	0.94–2.8	0.538	1.2	0.68–2.1
ECOG^C^ (≥2 vs. <2)	**0.000**	2.2	1.5–3.2	0.033	1.5	1.0–2.2
Stage (III/IV vs. I/II)	0.15	1.3	0.90–2.0	**0.01**	1.7	1.1–2.5
LDH^D^	**0.000**	1.1	1.0–1.2	0.114	1.4	0.92–2.1
NES^E^ (≥2 vs. <2)	0.322	1.4	0.71–2.9	**0.003**	1.7	1.2–2.5

^A^Hazard ratio, ^B^Hazard ratio 95% confidence interval, ^C^ECOG performance status, ^D^Lactate dehydrogenase, ^D^No. of extranodal sites.

Our signature was by far the most powerful independent prognostic factor. Significant *P* values were bolded.

### External validation of the prognostic gene signature

Next, the outcome predictor was checked to determine whether it could externally predict the outcome in the patients with DLBCL. Our results indicated that the developed signature was significantly associated with the clinical outcome of DLBCL in all the validation datasets, containing 417 patients, at a *P* value less than 0.0001 (Fig. 1). In GSE34171, our signature stratified the patients with distinct outcomes—with corresponding 5-year OS rates of 94% and 53% in the low-risk and high-risk groups, respectively. Additionally, in GSE4475, our signature divided the patients into 2 distinct outcomes—with corresponding 5-year OS rates of 60% and 20% in the low-risk and high-risk groups, respectively. In GSE11318, the rates of OS at 5 years in the low-risk and high-risk groups were 60% and 35%, correspondingly (Fig. 1). The hazard ratios for GSE4475, GSE11318, and GSE34171 were 0.32 (0.19–0.54), 0.51 (0.35–0.76), and 0.10 (0.02–0.45), respectively (*Ps* ≤ 0.001) (Table 2).

### Final prognostic signature and subtype of diffuse large-B-cell lymphoma

**Figure 2:**
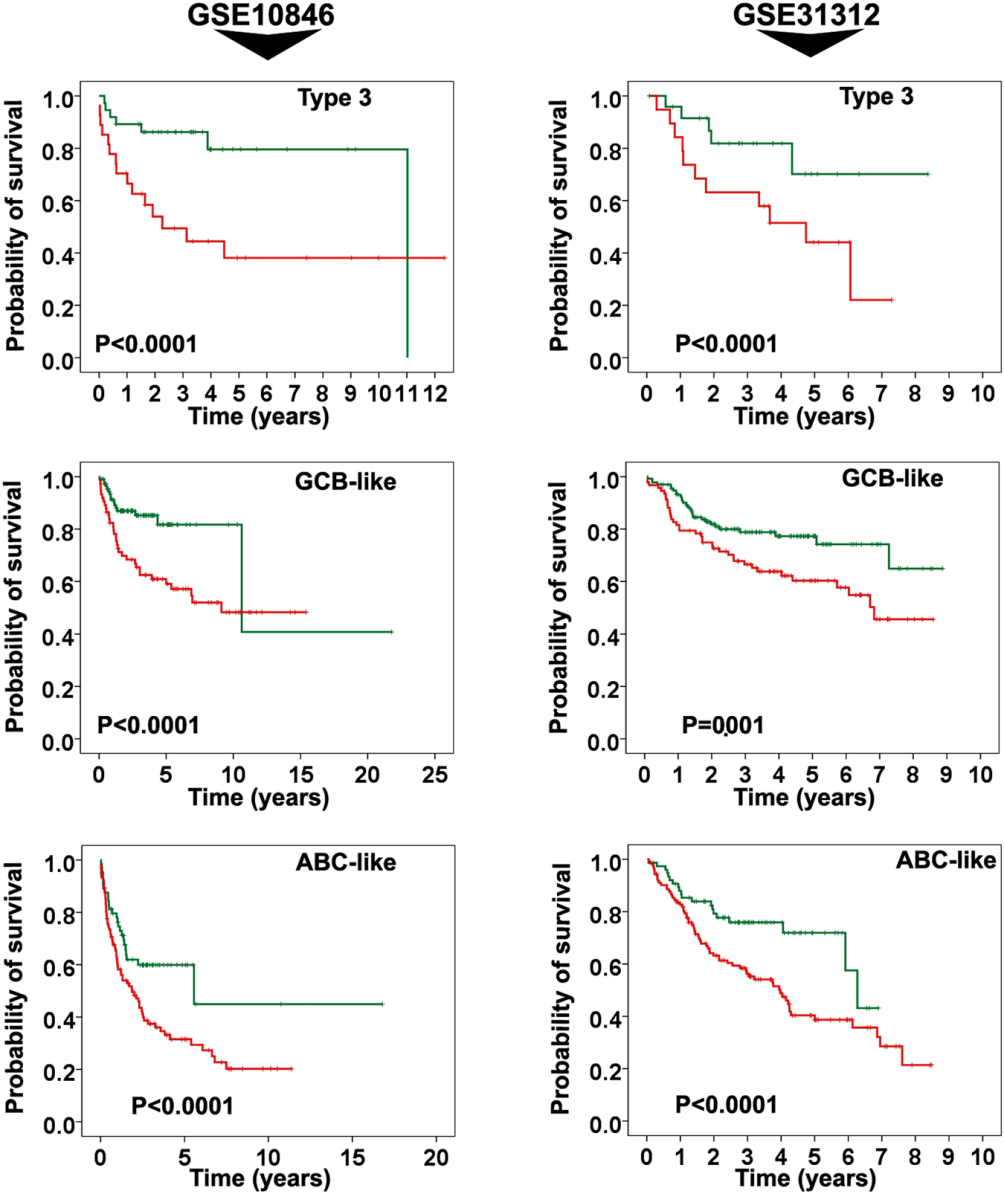
Kaplan-Meier survival analysis of the final 7-gene prognostic signature in three molecular subtypes of DLBCL (ie, ABC-like, GCB-like, and type 3). The survival time was significantly different between two risk groups constituted based on final signature in each molecular subtypes (*Ps* ≤ 0.001).

Our findings revealed that the survival time was significantly different between the 2 risk groups, constituted based on our signature, when applied in all the molecular subtypes of DLBCL—namely ABC-like, GCB-like, and type 3 (*Ps* ≤ 0.001) (Fig. 2). Hence, this outcome predictor was able to subdivide the patients within each subgroup into distinct risk groups.

**Figure 3:**
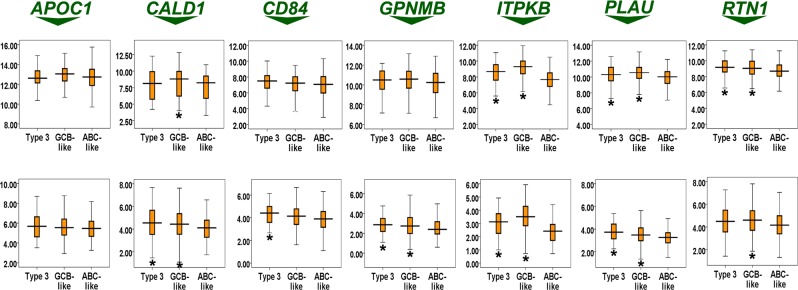
Comparison of the expression of our predictor components in three molecular subtypes of DLBCL (ie, ABC-like, GCB-like, and type 3). Upper and bottom panels indicate GSE10846 and GSE31312 datasets, respectively. Asterisk indicates significance compared with the ABC-like subtype (*P* < 0.05).

Our results also showed that the expressions of *CALD1*, *ITPKB*, *PLAU*, and *RTN1* were significantly diminished in the subtype with inferior survival (ie, ABC-like) compared with the subtype with better survival (ie, GCB-like) in both datasets (ie, GSE31312 and GSE10846) (*P*s < 0.05) (Fig. 3). In GSE31312, the expression of *GPNMB* was significantly lower in the ABC-like subtype than in the GCB-like subtype (*P*s < 0.05) (Fig. 3).

### Extraction of a revised prognostic gene signature from the final prognostic gene signature

**Figure 4:**
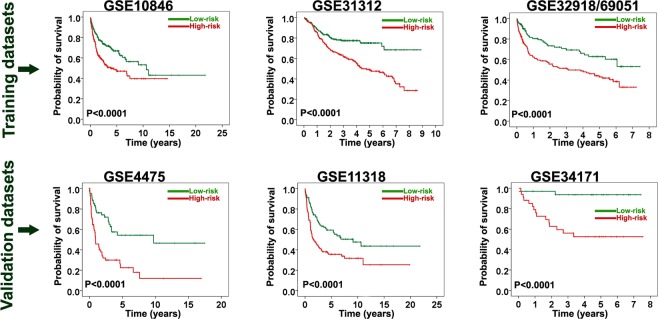
Kaplan-Meier survival analysis of the revised final prognostic signature in the training and validation datasets. The revised final signature was found to be significantly associated with overall survival at a *P* value < 0.0001 in both training and validation datasets.

The final signature was revised after the validation step. The goal of this step was to minimize the number of the genes to obtain a more practical signature which could be technically simple and applicable for routine clinical use. Our analysis showed that a combination of 3 genes—namely *APOC1*, *RTN1*, and *PLAU*—was able to divide the patients into high-risk and low-risk groups with distinct survival times in both training and validation datasets (*Ps* < 0.0001) (Fig. 4). The rates of OS at 5 years in the low-risk and high-risk groups for all datasets were approximately similar to ones in final prognostic signature (Fig. 4). Furthermore, the hazard ratios were significantly higher in the high-risk group than in the low-risk group (*Ps* ≤ 0.001) (Table 4). The hazard ratio of the revised prognostic signature was slightly higher than that of the final prognostic signature (Tables 2 and 4).

**Table 4:** Statistics of Cox proportional hazard analysis of the revised final prognostic signature in the training and validation datasets.

	*P* value	HR^A^	95% CI^B^
***Training datasets***			
GSE10846	**0**.**000**	0.57	0.42–0.78
GSE31312	**0**.**000**	0.46	0.34–0.65
GSE69051&32918	**0**.**000**	0.53	0.30–0.78
***Validation datasets***			
GSE4475	**0**.**000**	0.35	0.21–0.58
GSE11318	**0**.**001**	0.53	0.37–0.78
GSE34171	**0**.**000**	0.11	0.02–0.47

Significant *P* values were bolded.

^A^Hazard ratio, ^B^Hazard ratio 95% confidence interval.

Similar to the final prognostic gene signature, the revised prognostic signature was also independent of the IPI factors (*Ps* ≤ 0.001). This revised signature was by far the most powerful independent prognostic factor only in GSE31312 (HR = 0.47 [0.33–0.67]), but not in GSE10846 (HR = 0.61 [0.42–0.89]) (Table 5). In GSE10846, the hazard ratio of the revised signature was higher than that of the final signature in multivatiate analysis (0.61 vs. 0.39) (Tables 3 and 5).

**Table 5:** Multivariate analysis of the revised final prognostic signature and common prognostic variables in DLBCL (the IPI components).

Variable	GSE10846			GSE31312		
	*P* value	HR^A^	95% CI^B^	*P* value	HR	95% CI
Our signature	**0.000**	0.61	0.42–0.89	**0.000**	0.47	0.33–0.67
Sex (male vs. female)	0.824	1.0	0.67–1.4	0.118	1.3	0.93–1.8
Age (≥60 vs. <60 years)	**0.000**	2.1	1.4–3.2	**0.004**	1.7	1.2–2.4
**Molecular subtype**						
GCB-like vs. type 3	0.469	0.8	0.44–1.4	0.585	0.9	0.50–1.5
ABC-like vs. type 3	0.025	1.8	1.1–3.1	0.554	1.2	0.67–2.1
ECOG^C^ (≥2 vs. <2)	**0.000**	2.1	1.5–3.1	0.044	1.5	1.0–2.2
Stage (III/IV vs. I/II)	0.298	1.2	0.83–1.8	**0.006**	1.7	1.2–2.5
LDH^D^	**0.000**	1.1	1.06–1.2	0.059	1.5	0.99–2.2
NES^E^ (≥2 vs. <2)	0.716	1.1	0.57–2.3	**0.004**	1.7	1.2–2.5

Our signature was by far the most powerful independent prognostic factor in GSE31312. Significant *P* values were bolded.

^A^Hazard ratio, ^B^Hazard ratio 95% confidence interval, ^C^ECOG performance status, ^D^Lactate dehydrogenase, ^D^No. of extranodal sites.

### Evaluation of the prognostic genes of 3 previously published signatures in GSE10846 and GSE31312

As shown in Supplementary Table 2, except for *PLAU* and *ITPKB*, the majority of the other genes in the prognostic signatures proposed by Lossos *et al*.^2^, Rosenwald *et al*.^4^, and Wright *et al*.^6^ had no consistent associations with the survival time in the multivariate Cox analysis, where these genes were associated with long survival in one dataset and with short survival in another. Additionally, in case of consistent associations, the association was principally not significant in both datasets (*Ps* > 0.05) or it was significant in only 1 dataset (mainly GSE31312) (Supplementary Table 2).

## Discussion

In the present study, we sought to develop a gene-based prognostic predictor which could accurately predict the survival time in patients with DLBCL. Finally, we succeeded in constructing a 7-gene prognostic signature which robustly and reliably predicted the clinical outcome in our training and validation groups. As presented above, although the previously published prognostic signatures for patients with DLBCL can predict survival in their corresponding studied patients, they fail to predict the outcome in external groups of patients. Hence, we presumed that reconstruction of a prognostic signature from the genes commonly associated with the survival time in different groups of patients might resolve this problem. When mining the literature, we found that in studies with workflow similar to that in our investigation, an FDR below 10% or 15% was reasonable for the selection of significant genes. We mostly selected the common genes among genes significantly associated with survival with an approximate FDR of 0 (Supplementary Table 1), which means that the probability of a false positive was approximately 0.

We used the gene signatures of *APOC1*, *CALD1*, *CD84*, *GPNMB*, *ITPKB*, *PLAU*, and *RTN1* to reconstruct the final outcome predictor. Among them, *APOC1*, *GPNMB*, and *PLAU* were previously defined as members of the stromal-1 signature in a 108-gene model comprising 3 gene-expression signatures termed “germinal-center B-cell”, “stromal-1”, and “stromal-2” developed by Lenz *et al*.^8^. In addition, *ITPKB* and *PLAU* appeared in the outcome gene signatures of DLBCL proposed by Wright *et al*.^6^ and Rosenwald *et al*.^4^, respectively. Chiming in with our findings, these genes were associated with a long survival time in all these studies.

*APOC1* as an inflammation-related gene was found to be positively associated with the survival time in patients with DLBCL^7^. In addition, in breast cancer cells, this gene was regarded as an important tumor suppressor and cell proliferation inhibitor^8^. In contrast, it was reported that this gene was highly expressed in late-stage lung cancer^9^. Several studies have confirmed the potential role of *ITPKB* as an ideal tumor aggressiveness biomarker or favorable prognosis factor in DLBCL^6,10,11^. ITPKB (inositol-trisphosphate [IP3] 3-kinase B) was recently characterized as a critical tumor suppressor gene whose deficiency prompted DLBCL. Furthermore, ITPKB-activating agents can have curative potential^10^. This gene was among the gene cocktail used for the accurate categorization of DLBCL samples into ABC-like and GCB-like subtypes via a nuclease protection assay^11^. GPNMB (glycoprotein non-metastatic melanoma protein B) is highly expressed in different tumor cell types including glioma cells^12^, bone metastatic breast cancer cells^13,14^, low-metastatic melanoma cell lines^15^, and melanoma cells^16^. GPNMB was considered an important tumor suppressor in DLBCL^17^ and was reported to be differentially expressed in mantle cell lymphoma (MCL)^18^. *PLAU* (Plasminogen Activator, Urokinase) and *CALD1* (Caldesmon 1)—accompanied by *DCN*, *SPARC*, *FN1*, *MMPs*, and *PDGFRs*—are members of genes related to extracellular matrix remodeling^19^. Concurrent overexpression of *MMPs* and *PLAU* was associated with favorable prognosis in patients with DLBCL^4,7^. Additionally, overexpression of *PLAU* and *CALD1* was demonstrated in classical Hodgkin lymphoma tissues^19^. In contrast, high levels of *MMPs* and *PLAU* were associated with tumor invasion in some human solid tumors^20,21^. In our study, we found that *RTN1* was a favorable prognostic gene in both final and revised signatures. A previous study confirmed upregulation of *RTN1* in CXCR4^−^ DLBCL versus CXCR4^+^ DLBCL and reported that CXCR4^−^ and CXCR4^+^ subgroups were associated with a better and poorer survival time, respectively^22^.

Although we did not include *c5orf30*, *LPP*, *CSF2RA*, *PDLIM4*, and *RGS3* in our final gene signature, they can be considered single prognostic genes. Two of these genes—namely *CSF2RA* and *PDLIM4*—were previously determined as members of the stroma-1 signature, developed to predict the outcome of patients with DLBCL^7^.

In the current study, we developed a potential reproducible prognostic gene signature which was able to robustly discriminate low-risk patients with DLBCL from high-risk ones. In addition, we reconstructed a 3-gene signature from the final prognostic signature. Although the revised signature was not as powerful as the final signature, it was able to efficiently predict the outcome in both training and validation groups and was considered an independent prognostic parameter. Not only can these signatures be drawn upon in clinical approaches in tandem with other routine prognostic factors, but also they can be deemed molecular targets with a critical role in the biology of cancer.

## Methods

**Figure 5:**
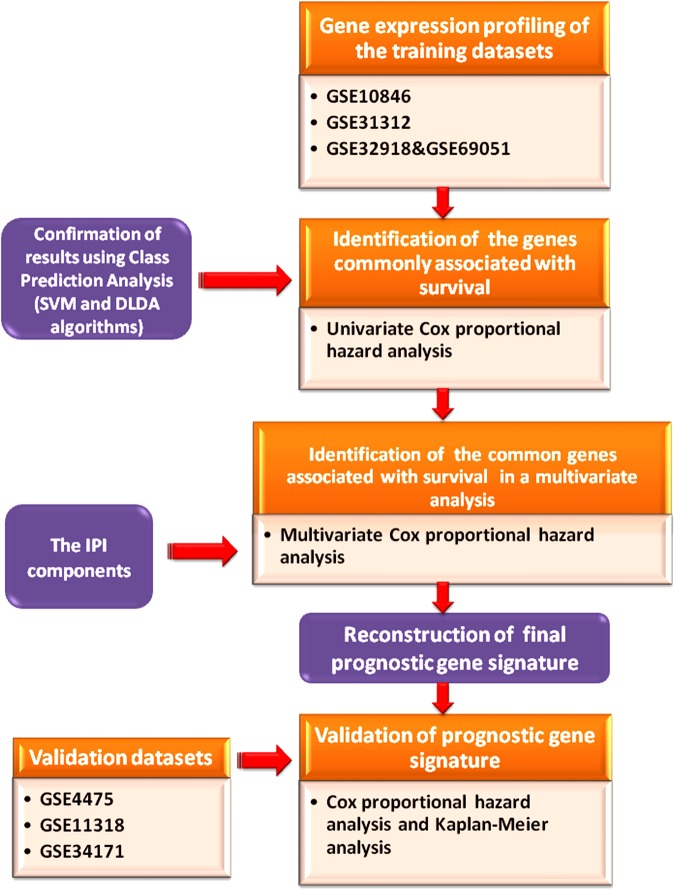
Schematic diagram depicting the analysis pipeline in this study.

A schematic diagram depicting the analysis pipeline in our study is presented in Fig. 5.

### Training and validation datasets

The Gene Expression Omnibus (GEO) (https://www.ncbi.nlm.nih.gov/geo/) database was searched to find the gene expression profiling datasets of patients with DLBCL. Only datasets containing clinical metadata (especially the survival time) (11 datasets) were retained, and the rest was excluded. Additionally, every effort was made to select expression datasets from all types of microarray chips such as Affymetrix and Illumina, if possible. The datasets were downloaded in the SOFT file format and were subsequently transformed logarithmically using tools provided in the geWorkbench 2.5.1 package^23^, if necessary. We employed various strategies to integrate different datasets used in our study. First, most of our datasets were generated using Affymetrix chip and GPL570 platform (Table 6). Hence, gene expression data were generated using similar approaches in these datasets. Furthermore, we only analyzed genes, which are existed in all chips and platforms. As another step, we normalize expression data using MAS 5 algorithm in all datasets. Hence, if a dataset were originally normalized using another method, we downloaded that dataset in raw format and then normalized it using MAS 5 method. More details on the clinical characteristics of the studied datasets are provided in Table 6. Some datasets with clinical metadata such as GSE57611, GSE23501, GSE93984, and GSE21846 were deleted for a specific reason (Table 6). The datasets were randomly divided into training (n = 1219) and validation (n = 417) datasets. In brief, GSE10846 (n = 420), GSE31312 (n = 470), GSE32918 (n = 172), and GSE69051 (n = 157) were used as training datasets, while GSE4475 (n = 123), GSE11318 (n = 203), and GSE34171 (n = 91) were utilized as validation datasets. Since GSE32918 and GSE69051 have originated from a similar research study^24^ and had some common samples, they were merged as a single dataset and named as GSE32918&69051. Number of samples for these datasets was determined after correction based on the common samples (172 samples for GSE32918 and 157 samples for GSE69051).

**Table 6:** Clinical characteristics of the microarray datasets used in our study.

Dataset	Number of patients	Chip manufacturer	Platform	Exclusion reason
GSE10846	420	Affymetrix	GPL570	—
GSE31312	470	Affymetrix	GPL570	—
GSE32918	172	Illumina	GPL8432	—
GSE69051	157	Illumina	GPL14951	—
GSE4475	123	Affymetrix	GPL96	—
GSE11318	203	Affymetrix	GPL570	—
GSE34171	91	Affymetrix	GPL570	—
GSE57611	37	Affymetrix	GPL96	FDRs of genes significantly associated with survival were above 95%
GSE23501	69	Affymetrix	GPL570	FDRs of genes significantly associated with survival were above 95%
GSE93984	60	Affymetrix	GPL570	FDRs of significant associated genes with survival were above 95%
GSE21846	29	Agilent	GPL1708	FDRs of genes significantly associated with survival were above 95%

### Identification of the common genes associated with survival in the training datasets

The association between gene expression and OS was examined using the univariate Cox proportional hazards analysis. In this analysis, the association between a group of covariates (genes) and the response variable (the survival time) was evaluated. The univariate Cox analysis was performed using the BRB-Array tools developed by Dr. Richard Simon and the BRB-ArrayTools Development Team. In this analysis, the findings were strengthened by employing a strict pipeline and retaining only genes with a *P* value less than 0.001 and a false discovery rate (FDR) less than 5%. Then, the common genes which were significantly associated with OS between the training datasets were extracted. For this purpose, only common genes with consistent associations were selected, while genes with inconsistent associations (negatively associated with OS in a dataset and positively associated with OS in another) were excluded. We also considered therapeutic regimens in the datasets used in our survival analysis. Hence, in each dataset, only common genes associated with the survival between patients with distinct treatments were selected for subsequent analysis. Additionally, for the confirmation of whether these genes were commonly associated with OS in all the training datasets, a class prediction analysis was also performed using 2 algorithms—namely support vector machine (SVM) and diagonal linear discriminant analysis (DLDA). In this analysis, 2 classes (ie, long survival [≥5 y] and short survival [<5 y]) were defined and thereafter classifiers, which could predict the 2 classes, were identified using 2 class prediction algorithms (ie, SVM and DLDA). The class prediction analysis was performed using the methods incorporated in BRB-Array tools.

### Reconstruction of the prognostic gene signature

The prognostic signature was developed as described previously^2,25,26^. In brief, the prognostic signature was reconstructed as a linear combination of the expression levels of the common genes and the z-score in the multivariate Cox regression analysis. Hence, at the first step, a multivariate Cox proportional-hazards regression analysis was performed for each gene, where all the individual components of the IPI (ie, age, stage, lactate dehydrogenase level, Eastern Cooperative Oncology Group [ECOG] performance status, and number of extranodal sites)^27^ and gene expression were entered as covariate variables. Additionally, sex and molecular subtype (ie, ABC-like, GCB-like, and type 3) were entered as another 2 variables into the multivariate analysis. The multivariate analysis was solely performed on the datasets with the clinical IPI data (ie, the GSE10846 and GSE31312). Afterward, the log-transformed normalized expression value of each gene was multiplied by the z-score. Finally, the prediction score was calculated for each patient as described in the following equation:

$${\rm{predictor}}\,{\rm{score}}={{\rm{z}}}_{1}{{\rm{G}}}_{1}+{{\rm{z}}}_{2}{{\rm{G}}}_{2}+{{\rm{z}}}_{3}{{\rm{G}}}_{3}+{\rm{\ldots }}{\rm{\ldots }}\,{{\rm{z}}}_{{\rm{n}}}{{\rm{G}}}_{{\rm{n}}}$$

Subsequently, the patients were first ranked based on their prediction scores before they were classified into 2 groups (>median value and <median value) and the survival times were compared between the groups using the Kaplan–Meier analysis and log-rank test at a *P* value less than 0.01. The survival analyses were performed using *Survival* (http://cran.r-project.org/package=survival) and SPSS 16.0 (Chicago, USA) packages.

### Evaluation of the prognostic gene signature in the validation datasets

The prognostic efficacy of the final developed gene signature was assessed externally in 417 patients as 3 validation datasets (GSE4475, GSE11318, and GSE34171). A workflow similar to the training datasets was performed. Similarly, the predictor score was calculated in the validation samples based on the details provided above. Subsequently, 2 groups were constituted after ranking patients based on their predictor score and then the survival time was compared between the groups using the Kaplan–Meier analysis and log-rank test at a *P* value less than 0.01.

### Prognostic signature and subtype of diffuse large-B-cell lymphoma

Whether the survival time was significantly different between the groups constituted based on our signature in each molecular subtype of DLBCL (ie, ABC-like, GCB-like, and type 3) was also investigated using the Kaplan–Meier analysis. Additionally, the expressions of the members of the outcome predictor were compared between these subgroups using the one-way ANOVA test at a *P* value less than 0.05.

### Extraction of a revised prognostic signature from the final prognostic signature

The goal of this step was to minimize the number of the genes to obtain a more practical signature which could be technically simple and applicable for routine clinical practice. Efforts were made to find a signature with a minimal number of genes, which could predict the patients’ clinical outcome with a statistical power similar to that of the final prognostic signature. To that end, in each round, 1 gene was deleted from the final signature and then the prediction ability of the remaining genes was tested using the Kaplan–Meier analysis and the log-rank test. A gene was considered a critical (hub) gene when its absence significantly reduced the prediction ability of the outcome predictor. Finally, critical (hub) genes were used to reconstruct a revised prognostic signature with the method applied for the final signature.

### Association between the prognostic genes in the signatures of Lossos *et al*. (2004), Rosenwald *et al*. (2002), and Wright *et al*. (2003) and overall survival in the GSE31312 and GSE10846 datasets

The ability of the prognostic genes in the previously published outcome predictors in the estimation of survival as well as the consistency of their associations with survival in the 2 big training datasets was evaluated by determining the association between the prognostic genes in the signatures of Lossos *et al*.^2^ (n = 6), Rosenwald *et al*.^4^ (n = 17), and Wright *et al*.^6^ (n = 14) and the OS time using the multivariate Cox proportional-hazards regression analysis in GSE31312 and GSE10846, as described above. Again, the IPI components and each gene were used as predictor variables and OS as the response variable.

### Ethical standards

Our study was performed using datasets deposited in GEO database. Hence, no ethical approval was required.

## Supplementary information

Supplementry Tables

## Data Availability

The datasets in the manuscript were deposited in GEO database (http://www.ncbi.nlm.nih.gov/geo/) with the accession number GSE10856, GSE31312, GSE69051, GSE32918, GSE4475, GSE11318, and GSE34171. Other supporting data are included as Supplementary Files.
