# Supplementary material for: Development of a Reproducible Prognostic Gene Signature to Predict the Clinical Outcome in Patients with Diffuse Large B-Cell Lymphoma
Source: Sci Rep. 2019 Aug 21;9:12198. doi: 10.1038/s41598-019-48721-0 (PMC6704056; doi:10.1038/s41598-019-48721-0)
Supplement: Supplementary file 1 — Supplementry Tables [file 41598_2019_48721_MOESM1_ESM.docx]

**Development of a Reproducible Prognostic Gene Signature to Predict the Clinical Outcome in Patients with Diffuse Large B-Cell Lymphoma**

Mohamad Zamani-Ahmadmahmudi, Seyed Mahdi Nassiri

| **Supplementary Table 1.** Statistics of the common genes associated with the OS in various datasets | | | | |
| --- | --- | --- | --- | --- |
| *GSE10846* |  |  |  |  |
| **Symbol** | **ProbeSet** | **P-value** | **FDR (x100)** | **Hazard Ratio** |
| APOC1 | 204416_x_at | 3.15E-05 | 0.00148 | 0.731 |
| C5orf30 | 221823_at | < 1e-07 | < 1e-07 | 1.955 |
| CALD1 | 201616_s_at | < 1e-07 | < 1e-07 | 0.672 |
| CALD1 | 201617_x_at | 2E-07 | 5.95E-05 | 0.742 |
| CALD1 | 201615_x_at | 1.3E-06 | 0.000193 | 0.827 |
| CALD1 | 212077_at | 2.2E-06 | 0.000275 | 0.707 |
| CALD1 | 214880_x_at | 5.71E-05 | 0.00211 | 0.765 |
| CD84 | 211192_s_at | 5.8E-06 | 0.000509 | 0.806 |
| CSF2RA | 210340_s_at | < 1e-07 | < 1e-07 | 0.722 |
| CSF2RA | 211286_x_at | 1E-07 | 3.47E-05 | 0.683 |
| CSF2RA | 207085_x_at | 2.6E-06 | 0.000307 | 0.784 |
| GPNMB | 1554018_at | 3.1E-06 | 0.000348 | 0.762 |
| GPNMB | 201141_at | 1.09E-05 | 0.000785 | 0.691 |
| ITPKB | 1554306_at | 1.21E-05 | 0.000822 | 0.769 |
| ITPKB | 235213_at | < 1e-07 | < 1e-07 | 0.756 |
| LPP | 202821_s_at | 2E-07 | 5.95E-05 | 0.792 |
| LPP | 202822_at | 0.000166 | 0.00395 | 0.711 |
| LPP | 235000_at | 3E-07 | 0.000124 | 0.628 |
| LPP | 224811_at | 0.000651 | 0.00984 | 0.75 |
| PDLIM4 | 211564_s_at | 1.47E-05 | 0.000916 | 0.831 |
| PDLIM4 | 214175_x_at | 3.47E-05 | 0.00154 | 0.815 |
| PLAU | 205479_s_at | 0.000492 | 0.00766 | 0.785 |
| RGS3 | 203823_at | 1.18E-05 | 0.000819 | 0.575 |
| RTN1 | 203485_at | 3.4E-06 | 0.00037 | 0.731 |
| RTN1 | 210222_s_at | 0.000167 | 0.00395 | 0.758 |
|  |  |  |  |  |
| *GSE31312* | | | | |
| **Symbol** | **ProbeSet** | **P-value** | **FDR (x100)** | **Hazard Ratio** |
| APOC1 | 204416_x_at | 0.000229 | 0.0186 | 0.772 |
| C5orf30 | 221823_at | 9.8E-06 | 0.00284 | 5.346 |
| CALD1 | 201617_x_at | 0.000001 | 0.000694 | 0.729 |
| CALD1 | 214880_x_at | 0.000004 | 0.00169 | 0.543 |
| CALD1 | 201616_s_at | 0.00089 | 0.0434 | 0.799 |
| CD84 | 211192_s_at | 0.000526 | 0.0315 | 0.738 |
| CD84 | 230391_at | 1.1E-06 | 0.000742 | 0.669 |
| CSF2RA | 211286_x_at | 1.40E-05 | 0.00494 | 0.571 |
| CSF2RA | 210340_s_at | 1.60E-05 | 0.00302 | 0.78 |
| CSF2RA | 207085_x_at | 0.000332 | 0.0352 | 0.754 |
| GPNMB | 1554018_at | 0.000763 | 0.0399 | 0.755 |
| ITPKB | 235213_at | 2.02E-05 | 0.00472 | 0.735 |
| LPP | 202822_at | 4.08E-05 | 0.00617 | 0.769 |
| LPP | 241879_at | 1E-07 | 0.000185 | 0.704 |
| PDLIM4 | 214174_s_at | 0.000859 | 0.0424 | 0.769 |
| PLAU | 211668_s_at | 1.81E-05 | 0.0308 | 0.747 |
| PLAU | 205479_s_at | 0.000112 | 0.049 | 0.792 |
| RGS3 | 203823_at | 9.41E-05 | 0.00459 | 0.799 |
| RTN1 | 203485_at | 0.000485 | 0.0299 | 0.789 |
|  |  |  |  |  |
| *GSE32918&69051* | | | | |
| **Symbol** | **ProbeSet** | **P-value** | **FDR (x100)** | **Hazard Ratio** |
| APOC1 | ILMN_1789007 | 6.1E-06 | 0.00725 | 0.572 |
| C5orf30 | ILMN_1677292 | 9.2E-06 | 0.00775 | 1.825 |
| CALD1 | ILMN_1717990 | 1.13E-05 | 0.00858 | 0.684 |
| CD84 | ILMN_2049293 | 0.000213 | 0.0368 | 0.595 |
| CSF2RA | ILMN_2376455 | 7E-07 | 0.00342 | 0.69 |
| CSF2RA | ILMN_1661196 | 2.84E-05 | 0.0146 | 0.661 |
| CSF2RA | ILMN_2376458 | 0.000255 | 0.039 | 0.783 |
| GPNMB | ILMN_1801205 | 7.67E-05 | 0.0227 | 0.67 |
| ITPKB | ILMN_1700432 | 0.000418 | 0.04 | 0.729 |
| LPP | ILMN_1651254 | 0.000746 | 0.0349 | 0.745 |
| PDLIM4 | ILMN_1663976 | 0.000901 | 0.0399 | 0.844 |
| PLAU | ILMN_1656057 | 2.7E-06 | 0.00509 | 0.664 |
| RGS3 | ILMN_1763371 | 0.000067 | 0.0219 | 0.53 |
| RTN1 | ILMN_1768357 | 0.000155 | 0.0306 | 0.693 |

| **Supplementary Table 2.** Analysis of univariate Cox proportional hazards analysis of the genes of signature proposed by Lossos et al (2004) (A), Wright et al (2003) (B), and Rosenwald (2002) (C) in GSE10846 and GSE31312. Green and red cells show positive and negative association with survival, respectively. Only *ITPKB* and *PLAU* was significantly associated with long survival in both datasets in Wright et al (2003) and Rosenwald et al (2002) signatures, respectively. | | | | | | | |
| --- | --- | --- | --- | --- | --- | --- | --- |
|  |  |  | **A** |  |  |  |  |
|  |  | GSE10846 |  |  |  | GSE31312 |  |
|  | HR (hazard ratio) | z score | P value |  | HR (hazard ratio) | z score | P value |
| BCL2 | 0.9439 | -0.86 | 0.3888 |  | 1.344 | 0.66 | 0.50914 |
| BCL6 | 1.17983 | 2.35 | 0.01861 |  | 0.214 | -2.91 | 0.00356 |
| CCND2 | 1.0378 | 0.72 | 0.46999 |  | 0.481 | -1.18 | 0.23989 |
| FN1 | 1.00796 | 0.17 | 0.86314 |  | 0.616 | -1.25 | 0.21305 |
| LMO2 | 0.9557 | -1.13 | 0.2584 |  | 0.1084 | -3.67 | 0.00024 |
| SCYA3 (CLL3) | 1.1795 | 1.74 | 0.08145 |  | 0.365 | -1.84 | 0.06507 |
|  |  |  |  |  |  |  |  |
|  |  |  | **B** |  |  |  |  |
|  |  | GSE10846 |  |  |  | GSE31312 |  |
|  | HR (hazard ratio) | z score | P value |  | HR (hazard ratio) | z score | P value |
| BCL6 | 1.17983 | 2.35 | 0.01861 |  | 0.214 | -2.91 | 0.00356 |
| CCND2 | 1.0378 | 0.72 | 0.46999 |  | 0.481 | -1.18 | 0.23989 |
| LMO2 | 0.9557 | -1.13 | 0.2584 |  | 0.1084 | -3.67 | 0.00024 |
| FUT8 | 1.2635 | 2.35 | 0.01852 |  | 0.1386 | -3.64 | 0.00027 |
| IGHM | 0.9575 | -1.09 | 0.27577 |  | 0.646 | -1.11 | 0.26767 |
| IL16 | 0.8864 | -1.12 | 0.26224 |  | 0.359 | -1.69 | 0.09119 |
| IRF4 | 1.1575 | 2.21 | 0.02706 |  | 2.171 | 1.53 | 0.12638 |
| ITPKB | 0.769 | -4.15 | 1.21E-05 |  | 0.735 | -3.49 | 2.02E-05 |
| LRMP | 0.9308 | -0.91 | 0.36176 |  | 0.267 | -2.75 | 0.00598 |
| MYBL1 | 0.917524 | -1.81 | 0.07045 |  | 0.76 | -0.67 | 0.506 |
| PIM1 | 1.1874 | 2.4 | 0.01659 |  | 1.94 | 0.57 | 0.5712 |
| PTPN1 | 1.00126 | 0.02 | 0.9843 |  | 0.413 | -1.23 | 0.21936 |
| CD39 (ENTPD1 ) | 1.19937 | 2.07 | 0.03861 |  | 0.1151 | -3.55 | 0.00039 |
| CD10 (MME) | 0.9334 | -1.65 | 0.09846 |  | 1.366 | 0.72 | 0.47393 |
|  |  |  |  |  |  |  |  |
|  |  |  | **C** |  |  |  |  |
|  |  | GSE10846 |  |  |  | GSE31312 |  |
|  | HR (hazard ratio) | z score | P value |  | HR (hazard ratio) | z score | P value |
| BCL6 | 1.17983 | 2.35 | 0.01861 |  | 0.214 | -2.91 | 0.00356 |
| FN1 | 1.00796 | 0.17 | 0.86314 |  | 0.616 | -1.25 | 0.21305 |
| BMP6 | 1.0684 | 1.04 | 0.29802 |  | 0.367 | -1.45 | 0.14619 |
| COL3A1 | 0.8766 | -2.42 | 0.01571 |  | 0.833 | -0.36 | 0.72018 |
| GCSAM | 0.8947 | -1.42 | 0.15589 |  | 0.303 | -2.41 | 0.01608 |
| GNL3 | 1.0948 | 0.74 | 0.45702 |  | 8.264 | 2.64 | 0.0082 |
| HLA-DPA1 | 0.9499 | -0.65 | 0.51802 |  | 0.271 | -2.07 | 0.03829 |
| HLA-DQA1 | 0.99266 | -0.29 | 0.76804 |  | 0.9414 | -0.26 | 0.7918 |
| HLA-DRA | 0.85123 | -1.38 | 0.1678 |  | 0.728 | -0.52 | 0.60397 |
| HLA-DRB1 | 0.74503 | -2.06 | 0.03943 |  | 0.458 | -0.84 | 0.40202 |
| MYC | 1.2143 | 1.78 | 0.07553 |  | 4.9503 | 2.51 | 0.01224 |
| NPM3 | 1.02274 | 0.21 | 0.83244 |  | 3.697 | 1.83 | 0.06725 |
| PIEZO1 | 1.2362 | 2.86 | 0.0042 |  | 0.635 | -0.58 | 0.5595 |
| PLAU | 0.785 | -2.9 | 0.000492 |  | 0.792 | -3.2 | 0.000112 |
| PPBP | 1.00066 | 0.02 | 0.98753 |  | 0.684 | -0.44 | 0.65851 |
| SERPINA9 | 0.935 | -1.75 | 0.07932 |  | 1.0358 | 0.05 | 0.96149 |
